# Key Enabling Technologies for Point-of-Care Diagnostics

**DOI:** 10.3390/s18113607

**Published:** 2018-10-24

**Authors:** Elisabetta Primiceri, Maria Serena Chiriacò, Francesca M. Notarangelo, Antonio Crocamo, Diego Ardissino, Marco Cereda, Alessandro P. Bramanti, Marco A. Bianchessi, Gianluigi Giannelli, Giuseppe Maruccio

**Affiliations:** 1 CNR NANOTEC, Institute of Nanotechnology, via Monteroni, 73100 Lecce, Italy; 2 Azienda Ospedaliero-Universitaria di Parma, via Gramsci 14, 43126 Parma, Italy; notarangelof@ao.pr.it (F.M.N.); antonio.crocamo@studenti.unipr.it (A.C.); dardissino@ao.pr.it (D.A.); 3 STMicroelectronics S.r.l., via Olivetti 2, 20864 Agrate Brianza, Italy; marco.cereda@st.com (M.C.); marco.bianchessi@st.com (M.A.B.); 4 STMicroelectronics S.r.l., via Monteroni, 73100 Lecce, Italy; alessandro.bramanti@st.com; 5 National Institute of Gastroenterology, “S. De Bellis” Research Hospital, via Turi 27, 70013 Castellana Grotte, Italy; gianluigi.giannelli@irccsdebellis.it; 6 Department of Mathematics and Physics, University of Salento, via Monteroni, 73100 Lecce, Italy; giuseppe.maruccio@unisalento.it

**Keywords:** lab-on-chip, point-of-care, in vitro diagnostics, biosensors, microfluidics

## Abstract

A major trend in biomedical engineering is the development of reliable, self-contained point-of-care (POC) devices for diagnostics and in-field assays. The new generation of such platforms increasingly addresses the clinical and environmental needs. Moreover, they are becoming more and more integrated with everyday objects, such as smartphones, and their spread among unskilled common people, has the power to improve the quality of life, both in the developed world and in low-resource settings. The future success of these tools will depend on the integration of the relevant key enabling technologies on an industrial scale (microfluidics with microelectronics, highly sensitive detection methods and low-cost materials for easy-to-use tools). Here, recent advances and perspectives will be reviewed across the large spectrum of their applications.

## 1. Introduction

The appearance of lab-on-a-chip (LOC) technologies and the improvement of micro total analysis systems (µTAS) have provided new tools for a broad range of applications, from health (diagnosis and disease management) to monitoring of environmental threats, as well as detection of bio-warfare agents, toxins and allergens in food and agriculture products. The interest in these platforms is worldwide, as witnessed by the international funding for research and the strong academic and industrial efforts to turn them into common use tools.

POC tests could in fact pave the way to personalized medicine in non-hospital settings, reduce the costs of health management, and even make the remaining hospital activity more agile and safer—e.g., decreasing the number of samples in laboratories reduces the risks of mislabelling and mishandling, and the consequent errors in results.

Today, unprecedented perspectives are opening up for the next generation of such devices. Important societal challenges will be addressed, e.g., in human health and environment preservation, through common-use tools for rapid and ultra-sensitive diagnostics and on-field testing assays (aka in vitro diagnostics—IVD or rapid diagnostic test—RDT). However, this goal requires intensive development of the relevant key enabling technologies (KETs). Among them, according to a classification, we should mention at least: advanced materials, nanotechnology, nano- and microelectronics, photonics, biotechnology and advanced manufacturing. This is, of course, a conventional division, as no KET can be treated as self-contained in an innovation strategy.

Personalized and preventive healthcare is the main target of the upcoming systems, which should be able to detect or monitor several relevant parameters, from blood pressure to biomarkers, both in clinical and domestic settings. Actually, this is the core idea of point-of-care (POC) diagnostics, whose applications, developed during the last decades, can be coarsely classified as (1) “near-patient” testing, for quick diagnosis and decision making or long run disease monitoring, and (2) on-field testing, to prevent the spread of epidemics or to test the safety of water and food.

Desirable features of POC devices or on-field assay, according also to FDA definition of “simple test” [1], include:

- Quick reliable response: A tests should last less than 1 h and the procedure should be as simple as possible, with few steps, and in compliance with the basic rules of good laboratory practice.
- Accuracy: sensitivity/specificity and detection limits should meet the legal limits needed for the specific application, improving or at least equaling the performances of traditional tests in order to enable medical decisions without further expensive tests so reducing impact on the public health costs. In this respect, nanotech-based approaches exploiting novel nanomaterials can provide new amplification methods for signal transduction with significant improvement in sensitivity. These include the use of metallic nanoparticles (NPs) or nanostructured metal layers for enhanced SPR or SERS analysis or as electrocatalytic labels as well as the use of nanowires, nanotubes and graphene [2,3].
- Ease of use: the test should be easily performed by unskilled people after minimal training, and the results should be clear and easy to understand.
- Self-containment: users should only be required to collect and deliver samples into the device. Reagent handling, analysis, data interpretation and storing of waste products should limit the intervention of users and their exposure to biohazard as much as possible.
- Portability and robustness: the tests should be carried out in the field, if needed, implying that they should be portable, resist the transport, and have a long shelf life. In the best cases, they should not even require electricity to work, neither cold storage.
- Low-cost: the platforms should be affordable for public healthcare systems, as well as for users and patients. The tests should be cheaper than standard, and should reduce the costs for the patient—for example in low-resource settings, where even the cost of travelling to healthcare structures could be discouraging.
- Multiplexing capacity: Multiplexed point-of-care testing (xPOCT), able to perform more than one analysis simultaneously [4], could enable a full characterization of a biological sample and a improvement in clinical diagnostics [5]—for example obtaining a complete molecular fingerprint of a patient allowing precision medicine approaches [5,6].

The development of POC diagnostic platforms with such characteristics requires remarkable efforts and a multidisciplinary approach across many technology areas. Below we will discuss some specific applications, as examples of the potentiality of POCs. In particular, we will first review the status of material research. Next, we will examine the most relevant innovative technologies aiming in particular to improve portability and shelf life, and to turn laboratory prototypes into commercial devices for on-field applications. Then, as a case study, we will present a test for drug sensitivity, based on a quantitative real-time polymerase chain reaction (qPCR) POC by STMicroelectronics. Finally, we will provide an insight into the global LOC market to understand the challenges that these technologies have to face to become commercially available.

## 2. POC Technologies in Low-Resource Settings and Developed World

In the developing world and low-resource settings, POC diagnostics could be invaluable for the quick screening of infectious diseases, which nowadays kill millions of people every year. Malaria, human immunodeficiency virus (HIV), tuberculosis and paediatric acute respiratory infections (ARIs) cause 95% of deaths due to infectious diseases all over the world. The situation is particularly dramatic in Africa, where access to medical care is not common and clinical treatments are often “syndromic”, i.e., based on the prevalent disease in that area. Related therapy, be it useful or not, often neglects the real disease [7]. Most hospitals are also overcrowded (only one or two doctors every 100,000 people, and these mainly in urban areas) and instruments for infection control are almost non-existent, since the contacts with infected persons are traced but not consistently isolated for monitoring [8]. Moreover, reaching hospitals could be challenging and expensive for people living far away. If RDTs could provide real time diagnosis, hospitals could discharge patients sooner, with an appropriate prescription, avoiding a second visit, with significant improvement in disease management. RDT devices for infectious diseases have been developed and marketed, but are still available for a restricted number of people. As an example, recently, Pollock et al. developed a paper-based POC fingerstick test for transaminase monitoring (particularly important in patients on therapy for HIV and/or tuberculosis). The test can determine the AST and ALT levels semi-quantitatively in 15 min [9], and the result is clearly identifiable to the naked eye as a change in colour (blue to pink for high AST, a red stripe for high ALT). The recent and running outbreak of the Ebola virus disease in West Africa, so large, severe and difficult to limit, is a dramatic consequence of the conditions of three of the poorest countries in the world—Guinea, Liberia, and Sierra Leone. The already precarious condition of these countries has worsened after years of conflicts. The civil war has left their national health systems largely destroyed or severely impaired. The outbreak has progressively become an international emergency and the scientific community worldwide is deployed to develop solutions like vaccines [10] or tests from biological specimens [11,12] to limit the crisis. In this scenario, the use of POC devices for mass screening of people would have considerably helped.

Another potential application of on-field assays is the detection of biological agents or toxic compounds from environment, a particularly important challenge in those areas where food and water are poorly controlled and checked. Once again, this could be the condition of many developing countries, where the native population often faces gastrointestinal diseases [13,14] and military forces working locally need to keep their personnel healthy and ready for operations. Multiparameter tools capable of detecting bacteria, DNA and RNA viruses, protozoans, toxins [15] and biowarfare agents [16] in food and water, would have a high impact on life-management.

Conversely, POC diagnostics is increasingly becoming of large-scale use in primary care settings in the developed world. Although often still administered by medical professionals, POC tests may be hopefully self-administered in some cases, making patients far more responsible for managing their own conditions. Tests for pregnancy and control of blood glucose concentrations are already of common use, but various emerging tools for more complex clinical or home management of diseases are also gradually spreading. Home POC testing reduces the frequency of hospital examinations, travel expenses, and loss of working time. Empowering individuals to test themselves can improve their compliance (adherence to diagnosis and treatment regimens) improving the clinical outcome. Portability/integration with “telemedicine” or “telehealth” ensures the medical supervision by giving healthcare professionals partial control over patient self-testing and data management, overall resulting in greater patient satisfaction. Recent studies published by Heneghan et al. report a 50% reduction in thromboembolic events among patients who self-monitored their International Normalized Ratio (INR, a prothrombin-related parameter useful in the management of heart diseases) using POC devices and adjusted their warfarin doses (oral anticoagulation drug [17]) using a nomogram [17,18,19].

The vast problem of cardiovascular diseases (like heart attacks and stroke) involving around 18 million people annually worldwide (considering the early symptoms) [20], is driving the cardiology diagnostic market. On-site POC tests for cardiac injury markers (myoglobin, creatinine kinase isoenzyme MB-CKMB) and cardiac troponins (cTnI and cTnT) facilitate effective screening, lower hospitalization rates, and cost saving. It is worth noting that, although cTnI and cTnT are the best validated, several other direct and indirect biomarkers such as myoglobin, ischemia-modified albumin (IMA), glycogen phosphorylase isoenzyme BB, copeptin (C-terminal proAVP), fatty acid-binding protein (FABP), B-type natriuretic peptide (BNP)—mostly measured as NT-proBNP—and myeloperoxidase have been identified in acute myocardial infarction (AMI) patients, and could be the targets of future RDTs.

Along with chronic diseases, the problem of cancer, whose global diagnostics market will reach $168.6 billion by 2020 [21], is driving the development of innovative devices, focusing on the detection of protein biomarkers such as the prostate specific antigen (PSA), platelet factor 4, and carcinoembryonic antigen. Multiparameter, rapid diagnostic tools could be effective and save the public and patient’s money with pathologies like prostate cancer, usually requiring several further testing. One of the main problems with it PSA, for example, is in its low specificity, although its detection in routine blood tests is the only parameter approved by FDA [22,23]. Thus, in case of altered PSA levels, further tests such as digital rectal examination (DRE), trans-rectal ultrasonography (TRUS) or biopsy are often recommended, which, however, are highly invasive and alter themselves the PSA, modifying the integrity of the gland. Even worse, the combination of DRE and total PSA levels yields unreliable results in two-thirds of all biopsied men [24]. A number of new candidate markers for prostate cancer are under investigation. The combinations between some of them can favour easier and more accurate diagnosis. In this perspective, a multiparameter easy-to-use tool [25] would be really effective as a large-scale screening of people against prostate pathologies, avoiding uncomfortable and expensive tests.

Another aspect to be considered in developed countries is the number of elderly people that is rapidly growing. The recent advances in key enabling technologies, and in particular the emerging sector of wearable devices, can provide new solutions going towards the perspective of assisted living and smart aging, and the realization of an intelligent and personalized medicine through the continuous monitoring and self-management of an individual’s state of health [26,27]. A number of examples can be given concerning wearable or implantable devices, most of wearable devices are based on sweat monitoring in order to control levels of glucose, electrolytes or other analytes in perspiration [28,29], saliva [30], tears [31] and others body fluids, exploiting the advantages of minimally invasive tools with smart materials and technologies.

## 3. POC Tools for Personalized Medicine

The strong interaction between biology/medicine and the digital technologies, with their ability to generate and manage a large quantity of data, is driving the transformation of traditional medicine into the so-called “proactive P4 medicine”. The acronym is for Predictive, Preventive, Personalized and Participatory according to Hood and Friend [32,33] who firstly recognized P4 medicine as the next big step towards improved wellness. Sub-targets of this great challenge are (i) to supply tools and strategies to quantify wellness and easily distinguish disease from well-being in individuals; (ii) to enable scientists to generate and analyse previously inconceivable large quantities of digital data; and (iii) to practice medicine also in non-hospital environments.

Easier, more reliable disease quantification would improve the follow-up—which, in many cases, cannot be precise enough over time—primarily because it would require unrealistic series of tests, such as frequently repeated biopsies. On the other hand, personalization accounts for the genetic uniqueness of human beings—differing by about six million nucleotides from one another—suggesting that each person should be treated in a targeted way, rather than as basing on a statistical average. In particular, proteins—which are the target for many drugs (protein kinases, cytokines, receptors, or their substrates)—express differently in different patients going through equal diseases. One of the main goals of personalized medicine is then to identify sets of disease-specific biomarkers and combine them with a robust technology, to allow clinicians to screen patients in subgroups and prescribe the most suitable drug at the correct dose, with maximal effectiveness and minimal potential for adverse effects [34].

Protein expression profile is just one piece of the large mosaic of the big data. Sources include “omic” information coming from a large audience of suppliers: genomics, proteomics, metabolomics, interactomics, cellomics, organomics, in vitro and in vivo imaging, and other high throughput indicators. An interdisciplinary approach, with a strong contribution from microfluidics and nanotechnology, would be the key point towards miniaturization, parallelization, automation, and integration of complex procedures in a simplified tool.

One of the crucial points of integration is data management (storage, validation and modelling) in order to convert the big quantity of information—the so called “data explosion”—into an exploitable outcome [35]. To this aim, Schneider et al. recently published a work dealing with a promising interactive assistance tool, called Drug Target Inspector (DTI), which may provide an overview of the datasets coming from genomic, transcriptomic and proteomic information in a user-friendly way. Deregulated pathways may be identified and selected according to their pharmacological responsiveness, and through easy access to further relevant resources and database entries (NCBI gene, GO, KEGG and STRING). By proposing possible treatment options via the detection of deregulated drug targets, the system could play a key role in tumour diagnosis and assessment of progression phases. DTI also maps the gene expression data onto the corresponding network nodes and enables visual assessment of how the downstream molecules might be influenced, so depicting also the potential effects of a drug administration. Moreover, it considers (epi)-genetic variations with a crucial role in tumour initiation and progression. Somatic variant data can be uploaded and classified according to their impact on the protein sequence (e.g., stop gained, missense, frame-shift). Thanks to Ensembl’s Variant Effect Predictor (VeP) database, variations can be investigated using DTI integrated genome browser. In this way, genes carrying mutations and genetic variations are identified, and can be exploited as to their potentially major effect on the tumour sensitivity to certain drugs [36].

Once all the parameters relevant to a major disease are known, the disease itself can be stratified into its major subtypes, to match each patient with the most effective drug for his/her disease subtype. In addition, if one could know the genetic variants causing useless or dangerous drug metabolic effects, and correct the problem with “re-engineered” therapies, new perspectives would open up. One of the main application fields would be the management of cancer diseases. It is well known that tumour tissues show large intra-tumour heterogeneity, changing in time and localization (varying from primary carcinomas to associated metastatic sites), which may foster tumour evolution and adaptation, and easily overcome therapeutic strategies [37]. In this respect, Liquid Biopsy (LB) (including circulating tumour cells—CTCs—mentioned below, as well as circulating tumour DNA,—ctDNA—and exosomes—EXOs) can provide information on tumour aggressiveness and improve the prognosis prediction, to support clinical decisions and monitor anti-tumour treatment effects without the needing of repeated biopsies. An obvious advantage is that LB just requires standard blood collection, which is easily repeatable during the progression of the disease—a factor of paramount importance [38]. Many efforts have recently been focused on the implementation of new methods to isolate, detect, quantify and analyse elements from LBs with lab on chip (LoC)-technology offering new possibilities and important advantages [39].

For complete diagnosis and deeper understanding of a disease, several aspects require investigation. Hence, different POC devices have to be developed or applied, to identify different types of biomarkers ranging from proteins and nucleic acids to whole cells.

### 3.1. POC Tools for Cells Identification

Blood cell count is important in clinical diagnostics because alterations in their number can discriminate healthy condition from pathological status. For this reason, several prototypes of hematic analysers have been proposed in the last few years. There are also several commercial POC devices for blood cell counting, such as HemoCue, that can count white cells inside a blood drop. The drop is inserted into the HemoCue microcuvette, containing dried reagents for cells lysis and staining. Photometric analysis quantifies the blood cells within minutes with precision comparable to bench-top instruments [40]. Other, more complex systems can distinguish white and red blood cells, and even their subtypes. One of the most significant is Chempaq XBC [41], able to measure the concentration of haemoglobin and count red and white cells classifying them as lymphocytes, monocytes, and granulocytes. Each sample is analysed in a disposable device, and counting and size measurement rely on impedance spectroscopy, while the measurement of haemoglobin is optical at two wavelengths.

The POC approach is feasible in several other fields, for increasingly challenging diagnostic tasks. One of the most promising areas is the detection of circulating tumour cells (CTCs), i.e., cells shedding in blood from the primary cancer site in very low concentrations (around one CTC per billion normal blood cells in advanced cancers) [37,42,43,44,45]. CTCs are useful biomarkers to deeply understand the progression and genetics of the tumour [46]. The interest toward this topic and the already mentioned LB [47,48] has increased remarkably during the last five years, as demonstrated by the huge hike in number of academic publications combining biological investigation and technological improvement. Many efforts aim to implement POC devices with features of accelerating analysis times and lowering costs. One of the challenging targets for on-chip investigation of CTCs is the enrichment of samples in CTC by separating and collecting them from other circulating cells, followed by automatic characterization. Concerning CTCs, the main limiting factor is their small number in patient blood and many efforts have focused on implementing new methods. We can distinguish two broad categories of technologies:

- Biochemical methods. Usually CTCs are distinguished from haematological cells using antigens expressed on epithelial cells only (e.g., EpCAM in the immunomagnetic Veridex CellSearch^®^ system for breast, colon, and prostate cancer). These methods are limited by CTC’s heterogeneity and the lack of universally approved tumour markers for affinity capture. Moreover, they are intrinsically biased by the positive selection induced by the capture system. There will be some cells, such as those undergoing epithelial to mesenchymal transition (EMT) (the most phenotypically aggressive), which will remain out of the analysis. In addition, the binding of antibodies to CTCs surface could induce phenotypical alterations, resulting in a misleading subsequent molecular studies.
- Physical methods are label-free and based on differences in physical properties such as size, shape, plasticity and electrical polarizability. In this case, no specific surface biomarkers are needed with a significant advantage. However, the physical properties of CTCs can overlap with those of residential blood cells and accurate techniques for CTC isolation are required.

As example of biochemical methods, we report the strategy implemented by Kurkuri et al. to improve capture efficiency of CTC based on a disposable microfluidic device realized by the plasma functionalization of polydimethylsiloxane (PDMS) and its conjugation with the anti-epithelial-cell adhesion-molecule (EpCAM) monoclonal antibody. The authors performed model studies on planar surfaces demonstrating high-grade immunospecificity of cancer-cell capture using NCI H69 small-cell lung cancer cells and SK-Br-3 breast cancer cells. Thanks to a fine tuning of the flow rate, they reached overall capture efficiency of 80 to 90% in cell-spiking experiments in phosphate buffer saline [49]. CTCs have also been separated from other blood cells in a tumour marker-independent manner.

Another interesting example of this biochemical approach is the Ephesia cell capture technology developed by the Viovy group. They recently optimized a method for CTC capture and genetic analysis exploiting magnetic particles functionalized with EpCAM antibodies. By applying a magnetic field in the device, magnetic nanoparticles self-assemble in the microfluidic channel and form a regular array of high aspect-ratio columns, able to capture cells of interest through antibody–antigen interactions. Such device demonstrated a capture efficiency above 90% for concentrations as low as a few cells per ml. After capture and visualization, bead–cell complexes were released and collected by switching off the magnetic field. Cells can be then lysed and analysed by real-time PCR or other molecular investigations [50]. On the other hand, among physical approaches the size-based CTC isolation methods take advantage of their dimensions: CTCs are bigger than normal blood cells. One of the simplest methods is based on filtration that can be realized in microfluidic structures by realizing pillars, microposts or micropores with different geometries. For example, samples enriched in CTC clusters, which are prognostic of poor outcome in some kind of malignancies, have also been obtained by Sarioglu et al. in 2015, through the fabrication of specialized traps able to capture even two-cell clusters under low–shear stress conditions. The idea was to place triangular pillars throughout the microfluidic channels. Two close pillars formed a narrowing channel, funnelling the cells into an opening, where the edge of the third pillar was positioned to bifurcate the laminar flow. As blood flowed, single blood and tumour cells diverted to one of the two streamlines at the bifurcation and passed through a 12 µm × 100 µm aperture. In contrast, CTC clusters were stuck at the edge of the bifurcating pillar. Using the so-called Cluster-Chip, authors identified CTC clusters in 30–40% of patients with metastatic breast or prostate cancer or with melanoma [51].

**Figure 1:**
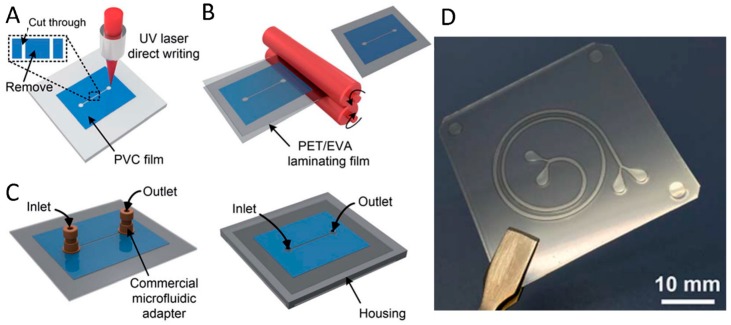
Schematic illustration of the fabrication process of the polymer film chip: the channel is realized by cutting a sheet of PVC film by UV laser direct writing (**A**); chip lamination by using PET/ EVA laminating films (**B**); two kinds of chip-to-world connexions; (**C**) Polymer film chip with a spiral microchannel (**D**). Reproduced with permission from [52].

An alternative tools was developed by Zhang and co-workers, and relies on a low-cost microchannel embedded in a polymer film chip (polyvinyl chloride, PVC), fabricated through UV laser writing and thermal lamination. The whole chip includes a spiral microchannel 500 µm wide and 120 mm long (Figure 1), allowing inertial cells isolation from spiked samples of human blood with good efficiency. Such a technique is able to separate CTCs on the basis of their different size [52].

Microvortices are useful to isolate cells on the basis of their size through inertial methods. Such devices consist of a series of expansion–contraction reservoirs within a microchannel in which the shear gradient lift force generate microfluidic vortices that can trap cells over a critical size in the center [53].

Both inertial microfluidics and filtration methods can be classified as passive methods since they do not require any external force. Some other methods classified as active methods require an actuation that can be electric [54] or acoustic [55,56] just to cite some examples. Dielectrophoresis (DEP) is one of the most used and more consolidated technique: it is based on the application of a non-uniform electric field. The DEP force depends on the size and dielectric properties of cells, so such a technique can be used to separate CTCs taking advantage from their differences in size and dielectric properties with respect to blood cells. One of the major troubles of DEP approach is due to bubbles formation caused by the direct contact with electrical connections. To overcome this limitation Sano et al. report an improved version of DEP devices by replacing metal electrodes with electrodes, isolated from the fluid in the main channel by a thin membrane, providing the electric field gradients for cell manipulation [57].

**Figure 2:**
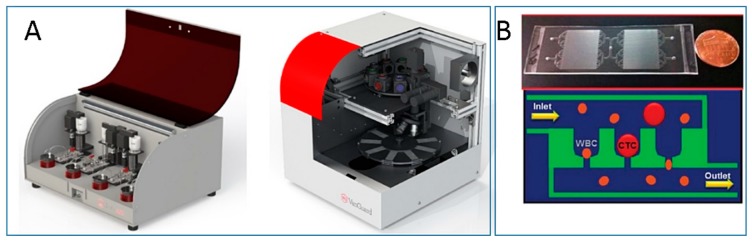
The “Celsee” system, able to process blood samples and perform image analysis (**A**). Microfluidic setup and scheme of the mechanism of CTCs capturing with inlet and outlet for pumping blood samples and reagents through the device (**B**). Modified with permission from [59].

Once isolated, CTCs have to be characterized. The related POC devices should then be integrated with molecular characterization tools, based on sensitive and low-cost methods [58]. A number of portable devices have been developed exploiting various detection systems. The platform realized by Mok and co-workers can easily test different types of proteomic biomarkers through simple electronics—that is, it should easily become a portable handheld device. Genomic studies have been performed on chip platforms as well, as described for example in the validation study recently published by Gogoi et al., in which the “Celsee” system facilitates rapid capture of CTCs from blood samples and characterizes them by immunohistochemistry, and DNA and mRNA fluorescence in-situ hybridization (FISH) (Figure 2) [59]. Portable setups have also been developed for nuclear magnetic resonance (NMR) characterization of isolated cells in on-field assays [60,61].

Besides cancer, other pathologies can benefit from personalized medicine and near-the-bed diagnosis approaches. The same approaches described for circulating tumour cells can be applied even for other type of cells such as circulating foetal cells or even for other analytes ranging from microvesicles and exosome to proteins.

Another field of application of POC devices is microbiology. For instance, a recently developed tool can detect three of the most common female genital tract pathogens directly from vaginal fluid. The platform is suitable for quick and low-cost screening of infections in the time of a gynaecological examination. This way, patients can leave the doctor’s office with a targeted antibiotic prescription, start the pharmacological treatment immediately, based on a real test and not only on symptoms, and without recurring to costly and time-consuming traditional assays like culture medium tests [62].

### 3.2. POC Tools for Protein Analysis

**Figure 3:**
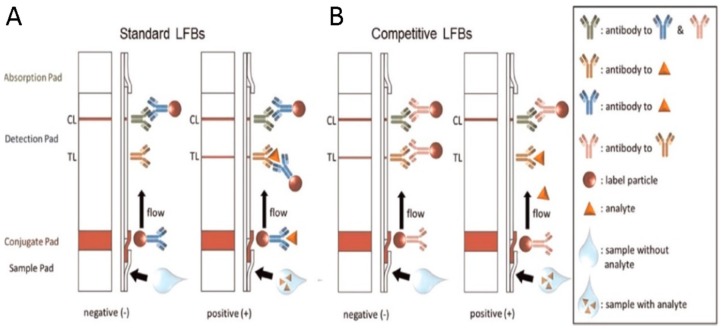
Scheme of a lateral flow test across with (**A**) standard and (**B**) competitive immunoassay. In the standard assay, when the sample is added the liquid start flowing to the conjugate pad where the analytes, if present on the sample, can bind to the label particles. The conjugate can flow by capillarity forces across the detection pad where they are captured only if the conjugate has the analytes attached. Instead, in competitive model the analyte and the label particles compete for being captured on the detection pad obtaining a response inversely proportional to the concentration of analytes. Reproduced with permission from ref. [63].

Proteins represent one of the major class of molecules used as biomarkers in POC assays. Compared to nucleic acid detection, which requires multiple steps of sample preparation such as cell lysis, nucleic acid purification, and DNA amplification, protein detection is relatively simpler, faster, and cheaper, thanks to analytical methods based for example on lateral flow or (immuno)chromatography. Most of the innumerable POC tests for diagnostics and self-testing are actually based on these two methods. Their low cost and quick response (around 15–20 min) make them the most widespread systems in both low-resource and non-laboratory environments. In addition, they are suitable for self-diagnosis and disease management. In lateral flow assays (LFAs), the separation of analytes flowing across a porous medium occurs thanks to specific interaction between antigen and antibody, enzyme and substrate, or receptor and ligand (Figure 3) [63].

**Figure 4:**
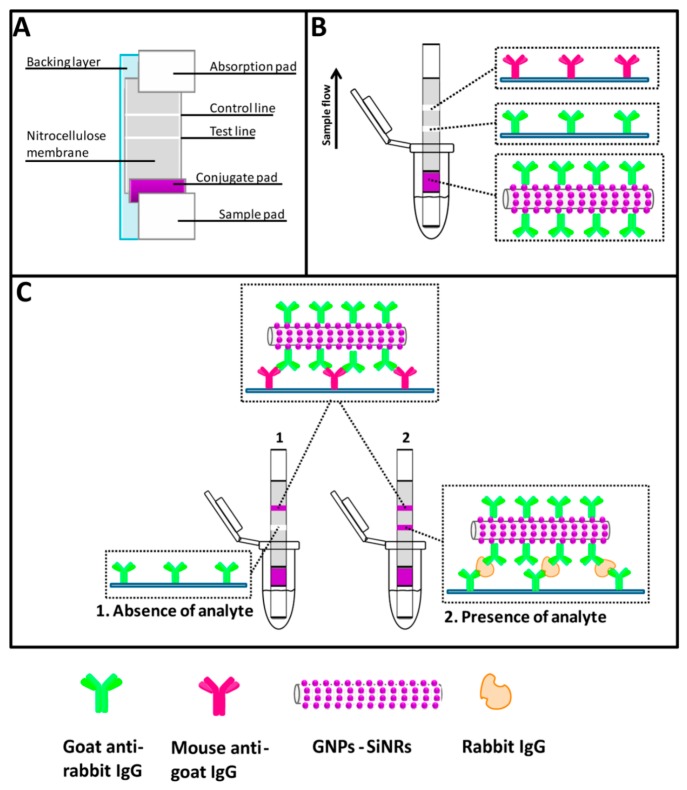
Lateral-flow strip biosensor described by Xu et al. (**A**) scheme of the device, (**B**) position of immobilized reagent on the strip and (**C**) measurement principle of the lateral-flow strip biosensor based on gold-nanoparticle-decorated silica nanorod (GNPSiNR) label in the presence and absence of rabbit IgG. Modified with permission from [68].

Usually the strip contains different areas, functionalized with various types of molecules, that specifically interact with the sample, producing colored or luminescent responses [64]. To increase sensitivity, the signal is often enhanced thanks to the use of nanoparticles (such as of gold [65], magnetite [66], silver [67]) conjugated with secondary antibodies, in sandwich-type immunoreactions yielding a colour signal. This is the case with the work of Xu and collaborators, who lowered the detection limit of simple gold nanoparticles-based assays by 50 times, using a gold-nanoparticle-decorated silica nanorod (GNPSiNR) label. GNPs on a single SiNR provided a purple color darker than the pure GNP solution (Figure 4) [68].

Besides diagnostic tools based on dipstick assay and lateral (or capillary) flow, others based on paper are often employed. Crucial aspects to be considered to optimize their operation are the surface characteristics, capillarity, porosity, and thickness of the paper. Paper, indeed, can be obtained from many raw sources such as wood (printing paper), cotton (filter and chromatography papers), jute, flax (linen), hemp, bamboo, and many others [69], with considerably different optical properties, porosity and surface chemistry. The latter two, in particular, critically affect the wetting properties and the behaviour of fluids on/in the device—and so, they may influence the overall performance. One of the most challenging goals is to obtain 2D or 3D microfluidic circuits and analytical setups directly on the “foil”, to allow transport fluids both horizontally and vertically, if required by the application. To obtain microchannels and define structures in paper, various approaches, including cutting, photolithography, plotting, inkjet etching, plasma etching, and wax printing have been proposed. Wax printing, for example, is rapid, inexpensive, and can selectively form water-repellent zones on filter paper thanks to its inertness to chemical reagents [70]. Rivas et al. recently improved the sensitivity of gold nanoparticle-based lateral flow assays for antibody detection, optimizing wax barriers (pillars) deposited onto the nitrocellulose membrane. Wax pillars created hydrophobic regions also in nitrocellulose membranes, with relatively fast flow. The controlled delays of the flowing fluid increased the binding time of the immunocomplex-detection antibody pair and generated a pseudoturbulence in the pillar zone, which would enhance the efficiency of the biorecognition event [71].

The lateral flow immunoassays (LFA) are widespread systems because of their low cost and quick response (around 15–20 min) but they often suffer from low sensitivity and lack of quantification. To address this issue LFA can be used in association with innovative reader systems that can improve LFA diagnostic performance in terms of sensitivity and contrast.

One possible solution, already on the market, is provided by QUIDEL that develops fluorescent detectors for LFAs strip. Such a system, called Sofia, is a small benchtop analyser based on an ultraviolet LED energy source for fluorescence detection: the optical sensor can collect hundreds of data points by scanning a LFA strip and automatically give an objective results [72]. Similar set-up have been commercialized even from other companies such as Qiagen who provides a reader system, called ESE-Quant Lateral Flow Reader for fluorescent and colorimetric detection of LFA strips [73].

Recently Wang et al. proposed a new method: their approach called thermal contrast amplification (TCA) is based on the laser excitation of gold nanoparticles. TCA reader is able to improve sensitivity (8-fold enhanced) and enable quantification in LFAs [74].

**Figure 5:**
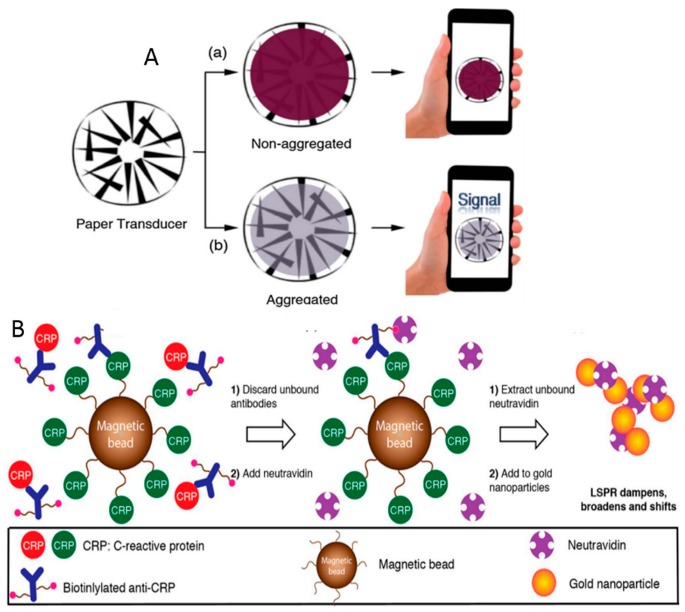
Schematic representation of the paper-based device developed by Russell and de la Rica. Modified from [75]. (**A**) A pattern is printed on filter paper; the presence of non-aggregated gold nanoparticles in suspension block pattern recognition the app, and no signal is generated; while aggregated nanoparticles do not impede pattern recognition (**B**) the methods is based the competitive immunoassay on magnetic beads that can cause the aggregation of gold nanoparticles. Modified with permission from [75].

As alternative to the LFA other optical tools have been developed: for example, an improved colorimetric approach on paper substrate has been proposed by Russell and De la Rica, based on localized surface plasmon resonance (LSPR) of gold nanoparticles (Figure 5). The authors demonstrated that patterns printed on paper can transduce LSPR variations caused by the aggregation of gold nanoparticles. The detector in this case was simply a smartphone camera and the proposed sensing strategy is based on triggering the aggregation of gold nanoparticles in presence of neutravidin. A competitive immunoassay has been applied to the detection of C-reactive protein. A common toner printer is enough to fabricate the transducers, while an augmented reality app for pattern recognition, running on a smartphone, can serve as the readout. Suspensions of gold nanoparticles blocks pattern recognition, while aggregations of nanoparticles do not, so generating a signal. This easy to use and cheap platform can be ideal to develop mobile POC biosensors for diagnostics [75].

**Figure 6:**
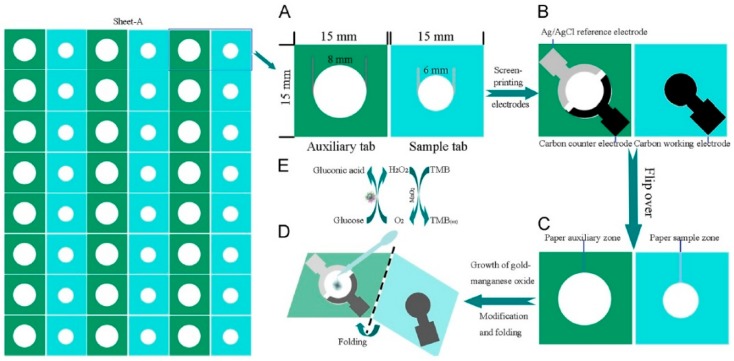
Schematic representation of the 3D origami device and assay procedure. Wax pattern of paper sheet (sheet-A) and (**A**) Single 3D origami device without the screen-printed electrodes. (**B**,**C**) Then electrodes were screen-printed on sheet-A and cut into individual 3D origami device. (**D**) After modification, the device was used to detect PSA exploiting an enzymatic reaction (**E**). Reproduced with permission from [76].

Paper-based devices usually take advantage of optical and colourimetric transduction methods. However, if on-paper microfluidics is available, other tools for analyte detection can be integrated. The device developed by Li et al. is based on amperometric transduction to detect PSA with a linear range of 0.005–100 ng/mL, and a limit of 0.0012 ng/mL. It uses glucose oxidase (GOx) as the enzyme label, tetramethylbenzidine (TMB) as the redox terminator, and glucose as the enzyme substrate. The authors grew a AuNPs layer on the surface of cellulose fibres, in a screen-printed paper working electrode (PWE). Subsequently, MnO_2_ nanowires were successfully electrodeposited on Au-PWE to form a 3D network with large surface area. Finally, the sample tab was folded down below the auxiliary pad, to keep the two parts of the device in contact, and then clamped to the electrochemical workstation (Figure 6) [76].

An alternative and valuable tool for point-of-care diagnostics can be LOC devices integrating electrochemical impedance spectroscopy (EIS)-based sensors. They respond to the need of fast response and low cost analysis, a major aim in clinical and proteomic tests. In this scenario, biorecognition events, as for example between antigens and antibodies (but can be even applied to complementary DNA strands), can be easily detected by EIS measurements, since the interaction of immobilized capture probes with analytes/targets molecules results again in a change in capacitance and interfacial electron transfer resistance. In particular, EIS biochips have been largely tested for the direct analysis of serum and biological fluids, being versatile and suitable for different functionalization protocols and demonstrating minimal interference from unspecific adsorption of biological components [77].

For example, the group of Maruccio and co-workers demonstrated application for on-chip diagnostics of prostate cancer, a disease largely diffused in the western male population and whose diagnosis is uncertain (if PSA serum concentration falls into the range of the so-called grey area) until a biopsy of prostate tissue is performed. The optimized platform allows a contemporary detection of free and total PSA thanks to the different functionalization and calibration of two chambers of the device, without recurring to expensive label-based standard techniques and providing an easily-processable electronic signal suitable for automated assays [25]. The same technology has been applied to the on-chip detection of other biomarkers for the diagnosis of pancreatic ductal adenocarcinoma, or allergens in food [78,79], as well as for on chip studies of cells’ behaviour [80,81,82].

### 3.3. POC Tools for Nucleic Acids Detection

Current methods for nucleic acid detection require expensive benchmark instrumentations and reagents, trained personnel and a long time, due to the multiple steps required (cell lysis, purification, amplification and detection of amplicons). The integration of all these steps in a chip-sized device can give new opportunities and overcome the current limitations.

Most of the techniques described for protein analysis have been applied even for nucleic acids detection but to achieve a real POC application additional functions have to be implemented. To this purpose, new devices for POC diagnostics should preferably perform not only detection but also sample preparation and molecular amplification. In turn, polymerase chain reaction (PCR) amplification, while seemingly simple, requires a refined technological approach, e.g., for precise temperature control. Alternatively, isothermal amplification methods such as loop-mediated isothermal amplification (LAMP), recombinase polymerase amplification (RPA) assays or helicase dependent amplification (HAD) assays can be used.

Centrifuge-based lab-on-a-disk is a promising technology to achieve on-chip DNA extraction. Several implementations have been proposed, achieving good spatial and temporal control over the fluid movement. For example, Choi and coworkers implemented a real-time fluorescence nucleic acid device for malaria detection, consisting in a compact analyser and a lab on a disk microfluidic chip. Magnetic actuation drove the manipulation of the sample. The rotation of the disk aligned different regions of the chip with an outer small magnet. Reagents were preloaded and separated by tooth-shaped passive valves. Each disk contained four parallel slots for simultaneous testing of four samples within 50 min. Each unit performed DNA binding to magnetic beads, washing, elution, LAMP reaction and fluorescent detection of amplicons [83].

**Figure 7:**
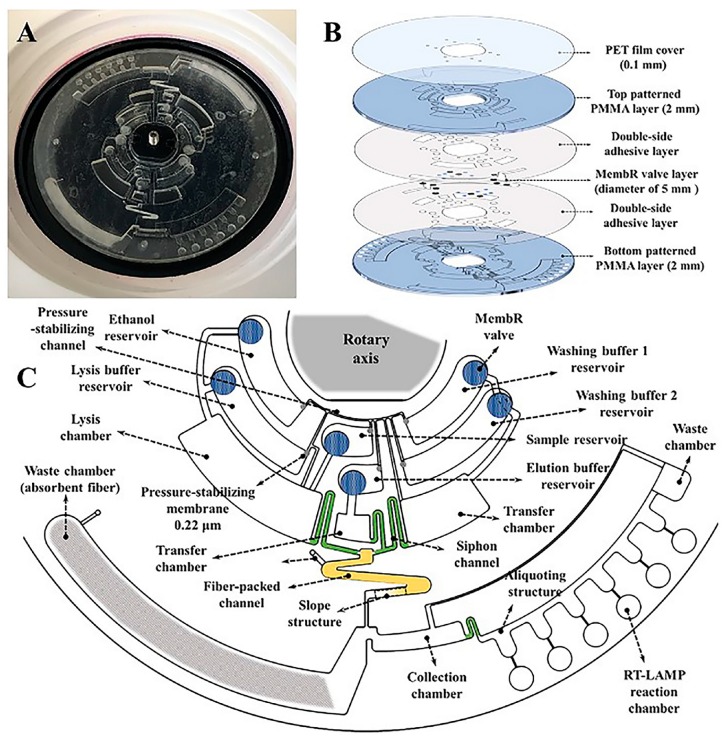
(**A**) Photograph of the assembled disc. (**B**) Assembly of the disc consisting of a PET film cover, a top patterned polymethyl methacrylate (PMMA) layer, a MembR valve layer, a bottom patterned PMMA layer, and two double-side adhesive layers. (**C**) Design of the diagnostic platform including six reservoirs with different solutions, a fibre-packed channel, an aliquoting structure, and six chambers for RNA extraction and RT-LAMP reaction; six different MembR valves, four transfer chambers and waste chambers. Reproduced with permission from [84].

An alternative approach has been described by Liu et al. who developed a novel lab-on-a-disk platform adopting membrane-resistance (MembR) valves for automatic fluid control. The MembR valves were realized using different polycarbonate membranes with superfine pore sizes, enabling pre-storage and manipulation of reagents under five different rotational speeds. With the help of MembR valves, all the steps from sample lysis, RNA extraction and purification, to amplification using real-time reverse transcription loop-mediated isothermal amplification (RT-LAMP) and RNA detection were integrated on the single device and applied to the detection of avian influenza viruses (HPAIVs). The whole set-up, controlled by a laptop, included accurate temperature control and weighed just 4 kg, in agreement with the requirements of a POC platform (Figure 7) [84].

**Figure 8:**
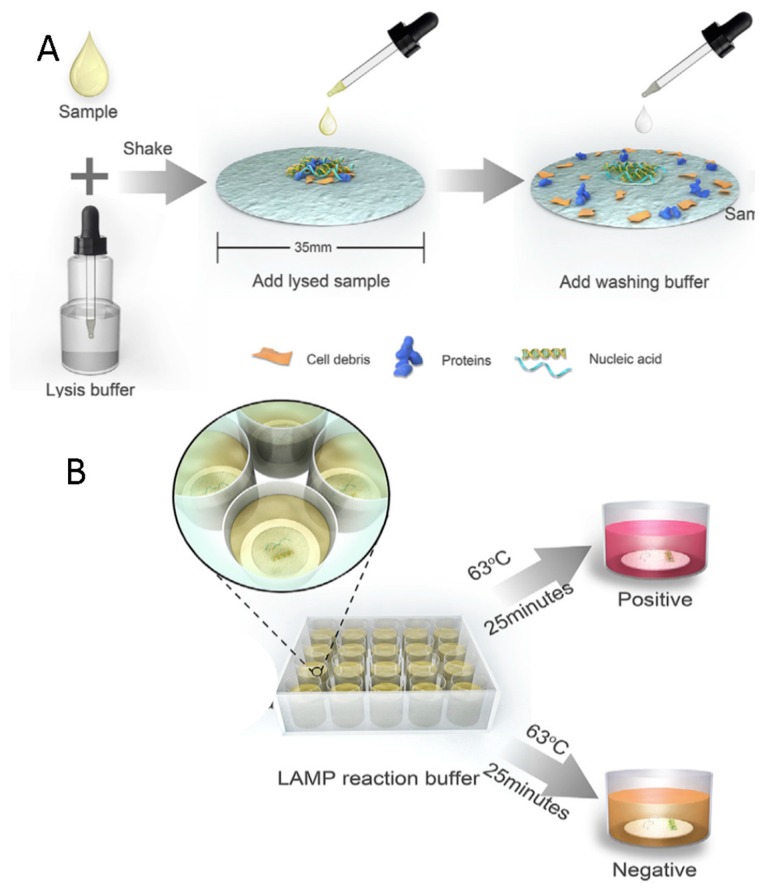
Schematic view of the paper based colourimetric assay proposed by Ye and co-workers: the lysed sample was added to the paper and washed with buffer, while nucleic acid are captured by the paper the contaminant of the lysed sample were eluted with washing buffer by capillary forces (**A**). Second, the sample adding area of the paper was cut and put in a micro-well for subsequent isothermal amplification. The nucleic acid captured on the glass fibre of the paper was directly used as the template for the high-efficiency LAMP reaction, and the results were visible by the naked eye on the basis of color (rose red positive for or brown for negative) (**B**) Modified with permission from [84].

Paper-based methods, previously described for protein analysis, are attracting great interest for nucleic acid detection too. Several prototypes have been realized through the integration of many functions required for nucleic acids analysis: extraction, amplification, and readout. For example, Ye and co-workers developed a paper-based, low-cost method that does not require any additional equipment for the POC diagnosis of rotavirus A. The test includes nucleic acid extraction, and subsequent amplification of the target sequences, at the end of which the amplicons could be visible to the naked eye or quantified by the UV-Vis absorbance (Figure 8) [85].

Among nucleic acids, miRNAs are of particular interest because of their key role in the development of several diseases like cancer [86]. Moreover, they are strictly related with patient-specific drug-resistance [87]. For these reasons, they have been carefully investigated, and are being increasingly considered as specific biomarkers with diagnostic, prognostic and theranostic potential. Potrich and co-workers recently reported the development of an innovative PDMS-based device able to selectively extract and adsorb extracellular miRNAs from cell supernatants, thanks to a specific functionalization of the polymer. The system implemented an original type of solid-state purification and adsorption of circulating miRNAs. The immobilized nucleic acids were then directly available for further reverse transcription into cDNA, through an on-chip system, without requiring detachment from the surface. The obtained cDNA was then analyzed via reverse transcription real-time quantitative PCR (RT-qPCR) to measure the expression rate of a specific miRNA in the extracellular medium [88].

**Figure 9:**
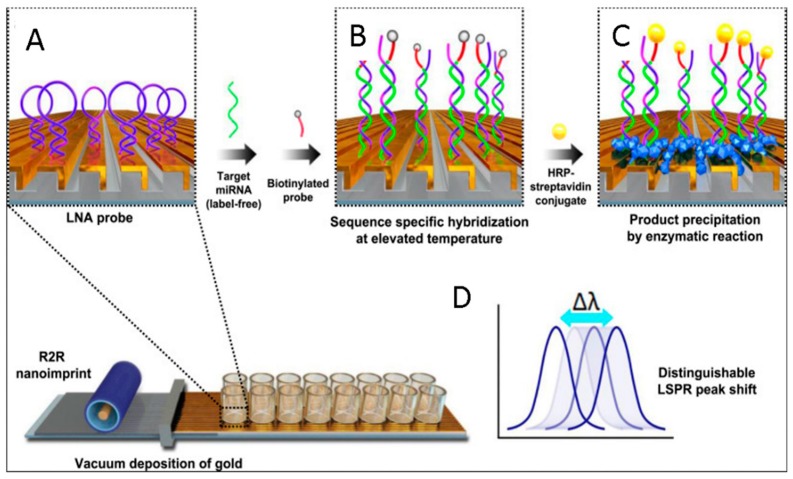
Schematic representation of a LSPR sensing platform based on flexible, transparent three-dimensional (3D) plasmonic nanostructure for the detection of miRNAs. A LNA hairpin probe is immobilized on the plasmonic structure (**A**). The hybridization with the specific miRNA cause the opening of the hairpin (**B**) and the subsequent binding of a second labelled probe for signal amplification (**C**). The presence of the specific miRNA can be detected by a shift of plasmonic peak (**D**). Reproduced with permission from [89].

Several other attempts have been made to integrate sensitive detection methods for miRNA quantification. A POC sensing platform for miRNA detection should enable label-free quantification with good sensitivity, real-time response, and high throughput. In this regard, Localized Surface Plasmon Resonance (LSPR) biosensors have attracted large interest, since they can be integrated into Lab-on-a-Chip platforms without the need for complex and sophisticated optical set-ups, unlike other sensitive optical techniques. For example, Na and co-workers proposed a sensitive LSPR-based miRNA sensing system based on flexible plasmonic nanostructures, fabricated by nanoimprinting, enabling single-base mismatch discrimination and attomole detection of miRNAs on real samples. They used a hairpin probe based on a locked nucleic acid (LNA). After hybridization with the specific miRNA, a second probe labelled with an enzyme induced signal amplification, forming a precipitate on the surface transducer through the enzyme reaction (Figure 9). Such a sensing platform may have important applications in POC diagnostics for detecting nucleic acids without the need for gene amplification [89].

Technologies for real-time quantitative PCR (qPCR) are reaching the market as POC tools for nucleic acids investigation, overcoming the needs of complex and expensive benchmark instrumentations. Two significant examples are the GeneXpert^®^ by Cepheid (already on the market) and the Q3 by STMicroelectronics (in industrialization phase) which are, to our knowledge, the two smallest instruments of their kind, while performances comparable with bigger instruments.

**Figure 10:**
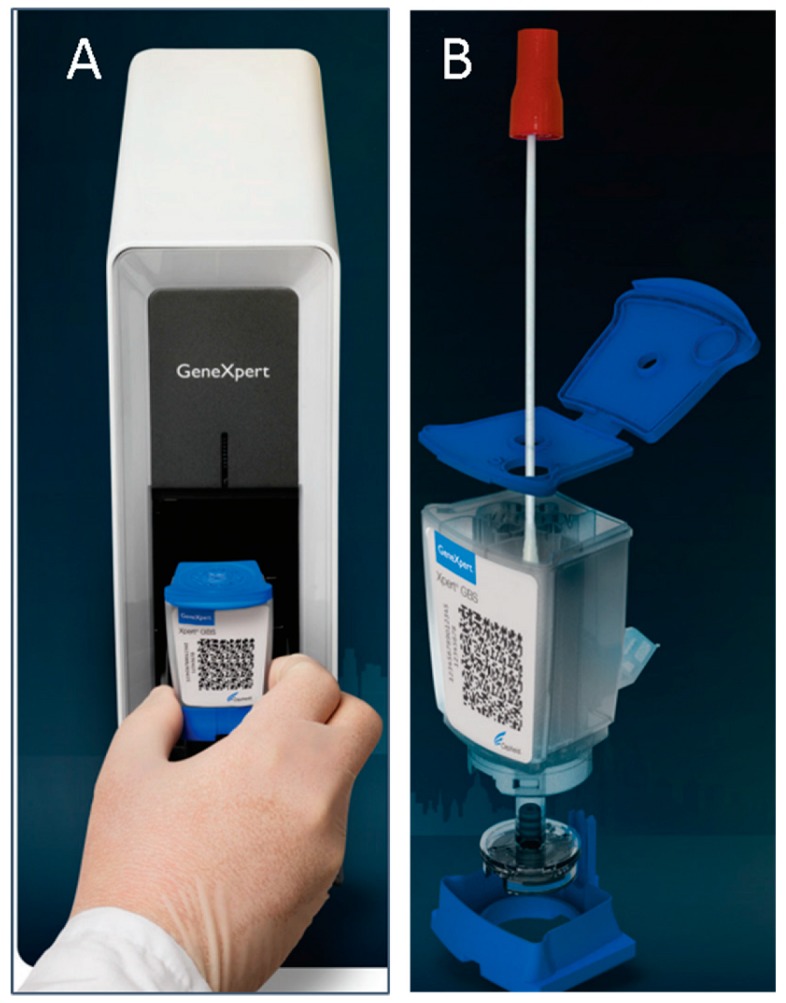
The Cepheid GeneXpert portable platform for qPCR. (**A**) The GeneXpert I model, including a single module able to run one cartridge at a time. (**B**) Exploded view of the disposable GeneXpert cartridge. The upper, bigger part includes the processing chambers where sample preparation occurs. Behind them (on the right in the picture) is the reaction tube where qPCR takes place. Below the processing chambers is the valve body, which drives all the fluidics. Figure modified from [93].

The GeneXpert^®^ by Cepheid (Figure 10) is probably the best-known and most mature POC qPCR system [90]. The GeneXpert I model in particular—that is, the single module instrument—is around 10 × 30 × 30 cm in size, and weighs around 8 kg. The reactions take place on a disposable cartridge, including a sample preparation system, so that the instrument manages an entire sample-to-answer flow, for the supported assays [91,92]. DNA is extracted by sonication-based cell lysis, then purified and mixed with the appropriate lyophilized qPCR reagents, to be eventually analyzed by qPCR. Each disposable cartridge contains one reaction chamber. Fluorescence detection relies on 6-channel optics. Multiple models are available with 1, 2, 4, 16, 48 or 80-module configurations for increased sample throughput.

**Figure 11:**
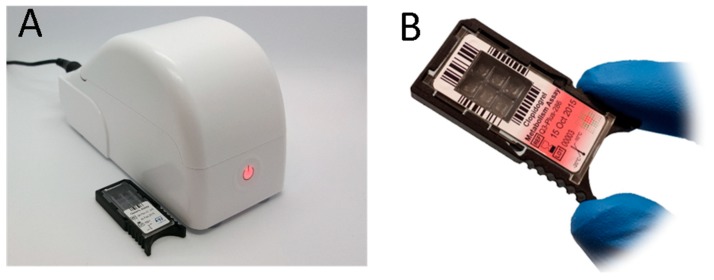
(**A**) The Q3 portable instrument closed, with its LOC cartridge next to it. (**B**) Front view of the Q3 LOC cartridge. Six reaction wells are visible—built over a silicon die—where six independent qPCR reactions occur.

On the other hand, the Q3 [94,95] is 14 × 7 × 8.5 cm in size and weighs just 300 g (Figure 11). The disposable cartridge is based on a silicon die—produced with established microelectronics technologies—and also hosts a printed heater + sensor pair, for precise temperature control. Each cartridge contains six reaction chambers, so that multiple tests can be run in parallel, possibly including, among others, replicate reactions, positive and negative controls, or even standard samples for on-board absolute quantification. Fluorescence detection relies on 4-channel optics. Q3 does not include any sample preparation system: the sample must be prepared outside and pipetted into the wells. However, it is general purpose and open, meaning that new assays can be built quite easily.

## 4. Innovative Sensing Elements for POC Applications

One of the biggest challenges of POC devices research and development is elongating the shelf life of tools containing biological probes as recognition elements. Apart from the obvious commercial advantage of a delayed expiring date (or the elimination of refrigerated transport and storage), innovation in the shelf life would make these technologies available in parts of the world where conditions (warm weather, war settings, extremely poor areas and so on) are problematic for standard clinical tests and POC diagnostics.

### 4.1. Molecularly Imprinted Polymers

One of the strategies towards non-perishable detection elements is the development of structures mimicking natural sensing elements but more resistant in order to eliminate the problems of refrigerated storage and transport. This is the case of Molecularly Imprinted Polymers (MIPs), which are cheap, being based on low-cost materials, and particularly versatile. Their biological applications range from sample purification [96] and compounds microextraction [97], to highly selective recognition of low-weight molecules [98,99]. Moreover, their binding sites can be regenerated, so enabling multiple reuse. Molecular imprinting implies the polymerization of precursors in presence of a template molecule, which is subsequently removed. Molecular cavities then form inside the synthetic polymer matrix, that are structurally and functionally complementary to the preselected template molecule or ion [100], featuring highly selective rebinding [101]. Furthermore, MIPs show remarkable stability under storage in dry state at room temperature, with a shelf life of several years without loss of recognition capability [102]. The integration of MIPs into biosensing platforms could follow various pathways, thanks to the possibility to mold polymers in the shape of nanoparticles [103], bulk [104] or thin layers over the electrodes or beads’ surface [105,106]. The use of MIP-modified sensors is recently spreading in the field of POC devices thanks to the easy control of film thickness and good reproducibility.

Recently, some sensors for cardiovascular diseases were developed based on MIPs: Moreira et al. in 2014 realized a low-cost disposable for rapid detection of myoglobin (Myo), a protein biomarker for Acute Coronary Syndrome. A screen printed electrode was modified with a MIP grafted on a graphite support incorporating a matrix composed of polyvinylchloride and o-nitrophenyloctyl ether as the plasticizer, followed by radical polymerization of 4-styrenesulfonic acid, 2-aminoethyl methacrylate hydrochloride, and ethylene glycol dimethacrylate with Myo as template molecule [107]. Also, cardiac troponin T (TnT) was detected with a high sensitive method based on electrosynthesis of poly(*o*-phenylenediamine) (PPD) film on gold electrodes by cyclic voltammetry. The rebinding capacity of the sensor was verified by cyclic voltammetry and impedance spectroscopy, for analysis of blood serum samples, amounting to a low-cost and useful tool for the quick diagnosis of myocardial infarction at the point of care [108].

MIP-based sensors were also developed for sepsis markers, with the ambitious goal of on-field diagnosis in low-resource settings, where sepsis is still one of the major causes of morbidity and mortality in neonates—causing 3.1 million newborn deaths each year [109]. The primary causes of sepsis include Group B Streptococcus (GBS) and *Escherichia coli* as the leading pathogens, accounting for over 60% of cases of early-onset sepsis [110]. Standard culture techniques can’t provide quick diagnosis, and even a few hour delay in antibiotic treatments may condemn to death sick patients. A number of strategies may be taken into consideration to develop new, rapid diagnosis tools. Buchegger et al., implemented a thermo-nanoimprinted biomimetic probe for immunosensing of LPS (lipopolysaccharide) and LTA (lipoteichoic acid), which are surface markers of Gram-negative and Gram-positive bacteria involved in the triggering of the inflammatory events during sepsis outbreak. To develop their assay, the authors pressed a LPS/LTA stamp onto a thermoplastic polymer thin film (Epon 1002F) with characteristics of high biocompatibility, derived from a liquid epoxy and bisphenol A. The MIP precursor solution was then transferred from the nanostructured PDMS stamp to the substrate via microtransfer molding. After photopolymerization, the stamp was removed leaving MIPs with specific target recognition. To investigate the ability of the imprinted polymer in rebinding the template molecules, the authors fabricated a quartz crystal microbalance (QCM) imprinted sorbent assays. Compared to the reference signal, the LPS-imprinted sites exhibited 13 times enhanced signals, while the LTA-imprinted sites resulted in a 3-fold signal enhancement, showing excellent rebinding capabilities of the thermo-nanoimprinted biomimetic probes [111]. A similar polymer nanoimprinting technique was used to modify the surface of an SPR substrate, for sensitive detection of Procalcitonin (PCT), another marker for sepsis. PCT molecules were firstly immobilized onto a glass support and kept in contact with a solution of 2-hydroxyethyl methacrylate (HEMA) and ethylene glycol dimethacrylate (EGDMA) deposited on a SPR substrate. Then, polymerization was performed and, after the removal of the PCT molecules from the polymer, specific molecular recognition sites were obtained, allowing a limit of detection (LOD) of 9.9 ng/mL from simulated blood plasma [112].

To further lower the costs and ease the handling and portability of MIP devices, Ge et al. recently developed a high selective “lab-on-a-paper” tool named MIP-based electro-analytical origami device (µMEOD) entirely realized on an A4 paper sheet. In the developed device, microfluidics connections were obtained by wax printing, and a carbon working electrode was screen printed on one of the folding parts. Gold nanoparticles were grown on the surface of the working electrode and MIPs for the chiral form of D-glutamic acid were grafted on the surface of particles for the detection of 0.2 nM of the neurotransmitter [113]. The growth of MIPs on the surface of nanoparticles has been exploited in various applications for the detection of low-molecular weight molecules. Magnetic [114] and silica-based [115] nanoparticles or quantum dots [116], covered with a MIP layer, have been used to improve the binding sites and the sensitivity of MIP-based assays. One of the most recent systems was implemented by Liu and co-workers, who made a coating of polydopamine on the surface of microbeads incorporating encoded multicolour quantum dots, thus implementing a multichannel detection method for the molecular recognition based on the absorbance spectra of encoded particles [116].

Another smart system suitable for high selective tests and miniaturization of components combines the MIP improvement in selectivity with the high-sensitivity of devices based on surface acoustic waves (SAWs). Basing on the analysis of acoustic waves at the surface of piezoelectric substrates, SAW systems can operate in the frequency range of 100−500 MHz, providing about an order of magnitude higher mass resolution than common QCM-based system, as the energy remains confined to the crystal surface, just where biorecognition reactions take place. Moreover, SAW devices are fully compatible with large-scale fabrication and multiplexing technologies and can allow implementing label-free methods for biosensing in liquid [117] and vapour samples [118]. A SAW/MIP sensor was recently developed by Maouche et al., who reached a LOD of 10 nM for Dopamine (DA) imprinted on a polypyrrole film, prepared by chronoamperometry electro-polymerization [119]. The sensitive detection of DA, a neuro-immunotransmitter in the central nervous systems of mammalians, is a parameter to detect the loss of DA-producing neurons, related to neurodegenerative diseases such as schizophrenia, Alzheimer and Parkinson’s diseases, or Tourette syndrome [120].

### 4.2. Lyophilized Reagents

Reagents such as antibodies for immunoassays or primer/probes and enzymes for nucleic acid detection may be stored in lyophilized (dried) form, to remain stable for a long shelf life without refrigeration, if controlled packaging preserves them from humidity. Based on this concept, POC tests were recently developed for the isothermal amplification and detection of Ebola virus based on freeze-dried reagents [121], as well as kits including beads made of lyophilized reaction components [122] for a rapid RT-PCR assay targeting the H1N1 Influenza A virus, that has periodically caused pandemics, due to frequent mutation of viral proteins [123]. Even if in the latter application the premixture reagents were stored at 4 °C, the authors implemented an innovative ready-to-use quantitative RT-PCR test, based on lyophilized beads including buffer salts, reverse transcriptase, AmpliTaq hot-start DNA polymerase and the primer-probe set. Each lyophilized bead also contained a passive reference dye for fluorescent signal normalization, and an internal control for PCR inhibitors’monitoring. The test consisted of lyophilized reaction beads organized into a ready-to-use 8-tube strip format. The beads could be completely dissolved in water within 5 s before use to detect virus infection in nasopharyngeal samples [122].

An alternative to freeze-drying methods is gelification of reagents, which Sun and co-workers optimized in a recent work [124]. All the necessary reagents were stabilized for long time storage by addition of gelifying and stabilizing agents, and desiccated at room temperature. This process minimizes liquid handling steps, allowing the reaction to start immediately upon rehydration with sample solution containing a DNA template. In particular, for this POC assay, PCR-based detection of the Campylobacter foodborne pathogen subspecies was optimized into the microfluidic circuits of a disposable polymeric lab card. Their Lab-on-a-foil exhibited a half-life at room temperature of at least 3 months without any alteration of the enzyme activity and long-term stability.

**Figure 12:**
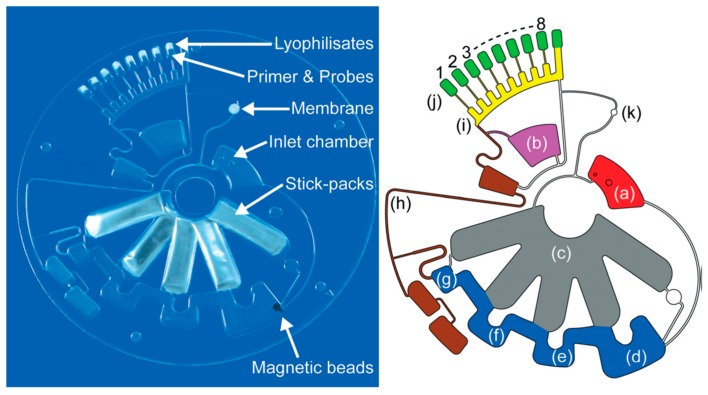
ab-on-a-disk platform for the automatic sequence of reactions for RT-qPCR with complete reagent prestorage. Photograph (**left**) and a CAD drawing (**right**) of the Lab-on- a-disk with the inlet chamber (a) stick-packs for reagents pre-storage (c) connected to the Teflon coated nucleic acid extraction structure (d–g) consisting of the lysis and binding chamber (d) wherein the magnetic beads are prestored, the washing chamber 1 (e) and 2 (f) and the eluation chamber (g). The microfluidic channels and pneumatic chambers in the area of (h) allow fluids handling to the aliquoting chambers (i) and to the reaction chambers (j) where primers, fluorescence probes and RT-PCR lyophilisates are prestored. Chamber (b) can be used. for loading a liquid RT-PCR mastermix instead of lyophilisates. reproduced with permission from [124].

An advancement in ready-to-use diagnostic tests for influenza A H3N2 was recently reported by Stumpf and co-workers, who implemented a “sample-to-answer” lab-on-a-disk platform for completely automated nucleic acid–based detection of respiratory pathogens (Figure 12). Its complex structure comprised microfluidics built with various techniques and materials: PDMS, cyclo-olefin polymer, Teflon associated with soft lithography, CO_2_ laser, ultra-precision micromilling machine. The circuit could hold the sample and deliver it through a circuit activated by centrifugal forces, applied according to a precise rotational protocol. Liquid buffers for nucleic acid extraction were pre-stored in miniature stick-packs, suitable for long-term storage. The stick-pack also contained frangible seals, which were opened during centrifugation by the liquid pressure reached at very well defined spinning frequency, and thanks to the presence of centrifuge-pneumatic valves. Among prestored reagents, authors included also air dried specific primers, fluorescent and magnetic conjugate probes, and lyophilized RT-qPCR mastermix. Employing two different release frequencies they achieved the on-demand stick-packaged liquid discharge of highly wetting extraction buffers, and the subsequent release of lysis and binding buffer. A strict running protocol was then applied to a prototype Lab-on-a-Disk player, able to finely tune the rotational speed and the applied magnetic field, so that reverse transcription and qPCR with real-time fluorescent readout were performed achieving a LOD down to 75 plaque forming units (pfu) per ml in a time for sample-to-answer of less than 3.5 h. The hardware setup was a 2 kg portable, laptop controlled, POC device [125].

### 4.3. Hydrogels

Hydrogels are another class of smart materials appealing for POC applications and suitable for integration in biodevices. Those employed for biological applications are usually made up of biocompatible polymers (e.g., acrylamide, acrylic acid, and its salts) and characterized by the capability of swelling and collapsing. Thanks to their molecular composition, rich in hydrophilic chains, once swollen, they can hold large amounts of water in their three-dimensional networks. Collapsing is induced upon a physical (light, temperature, magnetic field) or chemical (pH, ionic strength, solvents) stimulus [126] and causes the release of the same water. Thermoresponsive hydrogels are particularly suitable for POC tests to be used in warm countries, as the temperature of the transition state (LCST) can be easily tuned to higher values if needed. Changing the side chains, in fact, modifies the solubility of the polymer, resulting in a lower or upper critical solution temperature. For temperatures below the LCST, the hydrogel lingers in a swollen state, with a large amount of liquid incorporated into the polymer network. If the temperature increases above the LCST, the hydrogel collapses and the liquid is released.

Various chemical components, including many biomolecules, can be stored and released from thermoresponsive hydrogels [127]. Niedl and Beta recently combined paper-based microfluidic device with hydrogels to carry out complex fluidic protocols. The hydrogel was a thermoresponsive poly(*N*-isopropylacrylamide) (NIPAM) containing an 85% aqueous solution in the swollen state. Complete collapsing of the hydrogel was obtained within a narrow temperature window between 28 and 34 °C. Chemicals and enzymes were stored in dry conditions in the paper substrate, and dissolved upon liquid release from one of the hydrogel reservoirs. Depending on a different ratio between monomers, the hydrogel dissolution ratio could be tuned. Combining this feature with temperature modulation, the authors were able to deliver liquids with different flow speeds and thus dissolve the dried reagents, controlling the residence time of solutions in different parts of the device to optimize the reaction conditions [128]. Based on a similar principle, an aptamer-cross-linked hydrogel was used as a target responsive flow regulator in a paper-based device in the work of Wei et al. [129]. The aptamer was the cross-linker for the polymerization of a smart, target-responsive hydrogel, in which target binding could mediate gel−sol phase switching, suitable for portable and simultaneous detection of multiple targets, even in complex biological samples. If no target was present in the sample, the hydrogel would fill up the channel, stopping the flow and preventing the coloured spot produced by food dyes to appear. Conversely, when present, the target/aptamer recognition prevented the hydrogel formation, blocking the flowing of the indicator towards the observation spot.

In a recent publication, the advantages of molecularly imprinted polymers and stimuli-responsive hydrogel features have been combined into a fluorescent molecular gate, sensitive, water compatible and highly selective, capable of sensing the α-fetoprotein (AFP) at trace level. The stimuli-responsive fluorescent polymer matrix was synthesized by mixing glutamic acid derivative (with pH-responsive behaviour), a thermoresponsive monomer i.e., *N*-isopropylacrylamide (NIPAm) and a vinyl silane modified carbon dot, to enhance the luminescence of the imprinted polymer matrix. The fluorescence, in turn, was enhanced upon binding with template molecule (AFP). The fluorescence response was linear vs. increasing concentrations of AFP in the 3.96–80.0 ng/mL range, with a LOD of 0.42 ng/mL. The method was then faster, more sensitive and easier than the corresponding ELISA. The template binding to the MIP-cavities occurred if at least one among the temperature and pH were in the prescribed range [130].

## 5. Smartphone-Based Platforms

To improve the portability of smart detection systems for POC analysis, one of the most explored strategies is their integration with smartphones, which are almost ubiquitous in developed countries but can represent easily accessible interface even in developing countries [131]. The result is immediate feedback, allowing the patient to self-diagnose, enhancing the speed of life-saving tests, and supporting quick decision-making. Moreover, the dedicated apps for smartphone and tablets will increasingly contribute to science as “big data” sources, very useful to elaborate predictive models and decision algorithms.

**Figure 13:**
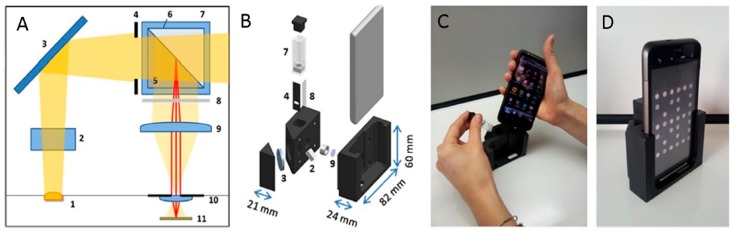
Optical (**A**,**B**) mechanical schemes of the Giavazzi et al. smartphone-based biosensing device. (**C**) Image of the assembled cradle during the insertion of the smartphone and the cartridge and the results of the analysis displayed on the screen of the smart phone (**D**). Adapted with permission from [132].

One of the recent examples of phone-based biosensing technologies is the work of Giavazzi and collaborators, in which the authors implemented a simple accessory turning a smartphone into a biosensor for label-free quantification of multiple markers (fractions of nM of blood markers for HIV and Hepatitis B in serum) within a few minutes and without requiring trained personnel. Sensing relied on a Reflective Phantom Interface, based on the measurement of the light intensity reflected by the surface of an amorphous fluoropolymer substrate. The latter featured a refractive index very close to that of the aqueous sample solution, and hosted various antibodies immobilized within spots. The light source was the phone flash LED, coupled with a tilted glass window for alignment with the sensor camera and directed to the bio-recognition surface. A diaphragm selected the portion of light illuminating the sensing surface of a perfluorinated prism, in contact with the sample solution in the measuring cuvette. The light reflected by the sensing surface passed through a polarizer and a converging lens, up to the phone camera hole, where one or more converging lenses were present, forming the image on the sensor. The CMOS sensor collected the reflected light through a mirror. The plastic cradle hosting the accessory sensor was made of three parts of black polyoxymethylene, holding the smartphone, measuring cuvette, magnetic stirrer and optical components. The system included also the phone’s autofocus device, which, after the cuvette was filled with aqueous solution, imaged the spotted sensing surface of the prism, then displayed on the screen (Figure 13) [132].

Another work based on the exploitation of phone components has been published by Liu et al., who demonstrated a portable fibre-optic surface plasmon resonance (SPR) biosensor employing surface electromagnetic evanescent waves at the metal dielectric interface. In this case, the smartphone SPR system employed a narrow-band filter placed between the flash of the cell phone and the lead-in fibers, providing nearly monochromatic incident light. Light interacted with the SPR-sensing region and was collected by the camera of the cell phone. Variation in the intensity of the light passing through the sensing elements was related to binding processes on the SPR sensor, quantifying IgGs at nanomolar concentrations [133].

The parallel advances in sensors and microfluidics together with the increased capabilities of the smartphone and the great efforts to integrate these technologies open new opportunities and applications for POC device. An interesting example is in the field of fertility investigation. For example, Kanakasabapathy et al. reported an automated smartphone-based platform for point-of-care male infertility screening to quantify sperm concentration and motility in semen specimens. The authors describe the integration of microfluidics, optical sensors, electronics, smartphone capabilities, allowing male fertility assessments in both developed and developing countries [134].

**Figure 14:**
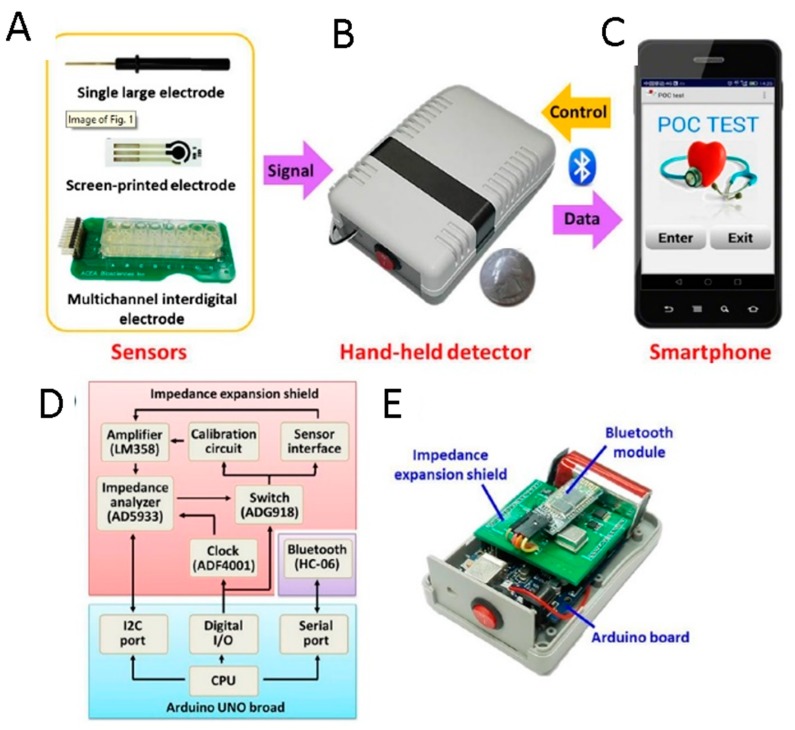
Smartphone-controlled electrochemical biosensor system realized by Zhang et al. The system includes electrodes (conventional large electrode, printed carbon electrodes, and interdigital gold electrodes) (**A**), a hand-held detector (**B**) and a smartphone controlling electrochemical measurements and feeding back signals (**C**) integrated in a circuit (**D**) by communicating with an impedance shield included in the hand-held detector through an Arduino board (**E**). Modified with permission from [136].

Recently, electrochemical sensors were integrated into a smartphone to detect molecules of clinical interest. In particular, a POC platform for the on-site detection of a protein from *Plasmodium falciparum* (the parasite causing malaria in humans) was reported including a microfluidic and electrical circuit interfaced with the phone through a USB host shield, and a printed circuit board to integrate the components for electrical communication and power distribution. The detection system employed a layer of antibodies against PfHRP2 parasite protein from human serum samples and required a tethramethylbenzidine (TMB)-labelled antibody, to exploit the peroxidase enzymatic product [135]. More recently, bovine serum albumin (BSA) was detected on the surface of printed electrodes through a smartphone-controlled electrochemical impedance spectroscopic analyzer. An Arduino board was the controller unit of the detector (receiving control commands from the smartphone through serial ports connected with the Bluetooth module). The smartphone in this case was used as a platform to deliver control commands, receive data signals, and display results of the electrochemical measurements in form of Nyquist plot. Finally, an Android app provided the interactive interface to the user (Figure 14) [136].

## 6. From Chip in a Lab to Lab-on-a-Chip—A Case Study

As an example, we report on the Q3 LOC device for qPCR, developed by STMicroelectronics, and its application to patients affected by Acute Coronary Syndrome (ACS) according to a protocol developed in cooperation with some Italian institutions—among them: Parma Hospital, Milan Niguarda Ca’ Granda Hospital, and Nuoro San Francesco Hospital; and the Universities of Milan and Parma.

ACS is a condition of impaired blood flow through the coronaries. The onset includes different signs and symptoms, and is often associated to myocardial infarction. Standard treatment for ACS patients includes antiplatelet therapy, associating aspirin to inhibitors of the ADP P_2_Y_12_ platelet receptors. Of the three inhibitors currently available—prasugrel, ticagrelor, and clopidogrel—the latter is the most diffused since, compared to the others, is cheaper and causes less bleeding. Its efficacy is however notoriously dependent on the patient’s individual response, in turn related to genetic variations of the CYP2C19 cytochrome P450 enzyme. In particular, in this highly polymorphic gene, *2 allele is the most frequent and causes of loss of function, with 15% frequency in Caucasians and Africans, and 29–35% frequency in Asians. In fact, while the so called “non-carrier” subjects of *2 allele are extensive clopidogrel metabolizers, those carrying one or two copies are intermediate and poor metabolizers, respectively. Conversely, carriers of the *17 allele are ultra-rapid metabolizers. Variations in the genes regulating clopidogrel absorption, such as *ABCB1*, may also influence the response to clopidogrel and consequent clinical outcomes. The bioavailability of clopidogrel is significantly reduced in carriers of the *ABCB1 3435* polymorphism, and homozygous patients are those exhibiting greater risk of adverse cardiovascular outcomes during treatment with clopidogrel [137].

These evidences led the US Food and Drug Administration to revise the clopidogrel instructions in 2010, mentioning the possibility of using the alternative treatments. On the other hand, although there is no consensus on the topic, some experts in the medical community support patient genetic testing for clopidogrel response, in order to provide different, more expensive therapies to poor metabolizers only [138].

Here we show an application of the Q3 portable instrument (14 × 7 × 8.5 cm, weighing 300 g), developed by STMicroelectronics—and already described in a previous section—to genotyping patients for clopidogrel response.

In a first phase, genomic DNA was extracted from 200 µL of peripheral blood from 160 ACS patients, and in 20 of them also from saliva. The Maxwell^®^ 16 platform (Promega Corporation, Madison, WI, USA) was used for both types of DNA extraction. qPCR analysis was then run on Q3, to detect the possible presence of three single nucleotide polymorphisms (SNPs): CYP2C19*2, CYP2C19*17 and *ABCB1 3435*. In parallel, all samples were also analyzed for the same three SNPs on an ABI PRISM 7900HT qPCR instrument (Thermo Fisher Scientific, Waltham, MA, USA), used as a gold standard benchmark. In addition, Sanger sequencing (on the ABI 3100 XI platform by Thermo Fisher Scientific) was applied randomly to 33 samples, to check the affordability of qPCR allele identification. The results from Q3 and the reference systems were 100% coincident, that is, the Q3 clinical specificity and sensitivity were 100% [94], enabling further studies.

In a second phase, a prospective, randomized, multicenter study was started. Patients were randomly assigned to either the pharmacogenomic group, or the standard care group. As to the former, peripheral blood samples from 448 ACS patients were collected and genomic DNA was extracted as said above. Then, qPCR analysis was run on Q3 platform only.

The study showed that a personalized approach to ACS with antiplatelet therapy selection, combining genetic information to standard clinical information, may improve clinical outcomes [139].

Q3 qPCR was always run in a 5 µL reaction volume, comprising 3 µL of reaction mixture and 2 µL of patient’s extracted genomic DNA. In turn, the reaction mixture contained 2.62 µL of TaqMan^®^ Fast Universal PCR Master Mix (Thermo Fisher Scientific) and 0.38 µL of TaqMan^®^ Drug Metabolism Genotyping Assay (a blend of two primers and two hydrolysis probes, FAM™– and VIC™–labelled, specific to one of the three SNPs to be detected; Thermo Fisher Scientific). The Q3 amplification protocol included an initial hold at 95 °C for 40 s, followed by 40 cycles at 95 °C for 3 s and 63 °C for 25 s.

**Figure 15:**
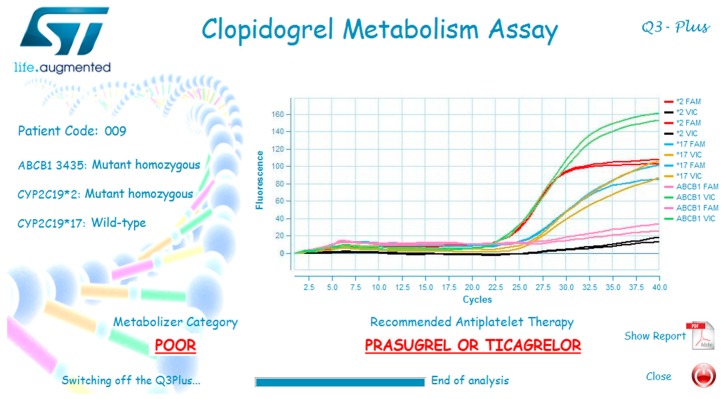
The Q3 software dedicated to the analysis of patients’ genotype for clopidogrel response. At the end of the analysis, the software clearly shows the diagnostic interpretation of raw qPCR results—in this case, which drug to administer to a patient, depending on her/his genotype.

Some significant Q3 results are reported in Figure 15. A Q3 analysis just requires a small quantity of patient’s DNA loaded by standard pipettes into the cartridge wells, since the qPCR reagents are pre-loaded into Q3 cartridges. The software developed for this application has all the reaction parameters embedded, and gives a clear diagnostic interpretation of raw qPCR results—namely which ADP P_2_Y_12_ inhibitor to administer. The overall analysis time (around 70 min, more or less equally divided between DNA extraction and Q3 qPCR) makes this technique suitable for real-time medical decision—also considering that, to be effective, the antiplatelet therapy should start within few hours from the early symptoms. In a near future, the DNA extraction phase could be in turn automated—and preferably integrated with Q3—into a sample-to-answer portable platform. All these features, along with the compactness and lightness of the system, could enable to run a test hopefully inside any emergency room, or even on board an ambulance, without the need of involving the hospital’s analysis laboratory.

## 7. Market Challenges

LOC systems have just begun to make their way in medicine, where they will likely achieve a prominent role in the next decades. The potential applications are so variegated that their full impact may not be easily appreciated at this point.

One of the crucial segments is molecular diagnostics, targeting one organism’s genome or proteome. When the target is in the patient’s genome—or proteome—molecular diagnostics opens the way to a personalized approach to medicine. An example has been presented in the previous section. PCR is nowadays the most widely used technology in molecular diagnostics, but other techniques are also emerging, like DNA sequencing, which represents one of the fastest growing application segments. According to some predictive studies, the molecular diagnostics market is projected to reach USD 11.54 billion by 2023, from USD 7.10 billion in 2017, at a stunning Compound Annual Growth Rate (CAGR) of 8.4% from 2017 to 2023 [140]. Other market analysts predict an even higher CAGR over the 2018–2024 period, reaching 12.1% [141]. This value is, presumably destined to grow even more strongly in the years to come, in an unpredictable and easy to underestimate fashion. As a matter of fact, the value of molecular tests depends on how much we know about the genome and proteome, and the clinical significance of particular targets for various pathologies—above all cancer, but also infectious diseases—or as to drug sensitivity, like for clopidogrel. The restless research on these topics will point out many other significant targets, and the specific tests that will be developed, will run on molecular diagnostics LOC platforms. The advantages of having them readily available in emergency rooms, medical offices, or even at pharmacies are evident: low-cost tests based on disposable and customized cartridges can be run when and where needed, without involving the centralized laboratories that nowadays perform molecular diagnostic tests—with a consequent decrease of all costs (including those for heavy benchtop instruments and specialized personnel required).

Besides the constant increase of scientific knowledge, the introduction of technological advancements in LOCs in terms of accuracy, portability and cost effectiveness is also expected to serve this market as a high impact driver.

Despite its huge importance, the field of molecular diagnostics does not gather all possibilities of LOC devices. Also more common types of medical analysis—such as standard blood tests—could be automatically performed even out of specialized environments, with remarkable advantages for patients in terms of comfort (small blood samples), lower cost and higher speed. Moreover, they could be either generic or targeted at a particular subset of values—e.g., blood elements count—for specific conditions requiring frequent monitoring of specific parameters. In the latter case, even home analysis could be conceivable, such as glucose monitoring that diabetic patients have to periodically perform and which, in this respect, are among the forerunners of home LOC devices.

In order to reach such capillary spreading, two technological steps are needed. First, more on-chip testing techniques must be developed and validated. The task is far from trivial, since a number of analyses can be performed on biological samples (not necessarily blood) involving variegated—mechanical/chemical/thermal—manipulation procedures, as well as different—optical/electrical—read-out techniques. Thus, while many molecular diagnostic techniques—including qPCR—use the same thermal control processes and fluorescence measurements to implement a huge variety of tests differing from each other only in the biochemical part, many other types of test will require ad hoc development of the instrument hardware—and the corresponding LOC in turn—although some building blocks may be recurrent.

On the other hand, in many cases, like qPCR, an initial sample preparation step is needed. Thus, all molecular diagnostic LOCs should be integrated with sample preparation subsystems capable of extracting DNA/RNA from the raw specimen through cell lysis, and separating it from waste—as already happens on some of the systems described above. This would allow an unskilled user—such as a patient with no specific knowledge—to manage the entire “sample-to-answer” flow. If these features still lack, laboratory equipment is needed in orderd to extract DNA/RNA with standard reagents and instrumentation and pipetting samples into the chip wells. These aspects still limit the use of most LOC devices to somehow specialized environments. The same considerations are valid for many other analysis techniques to be integrated on chip; and the number of combination of techniques for sample preparation with those for analysis pave the way to a plethora of possible applications.

Compliance to regulatory processes may also be a challenge in the massive diffusion of LOC devices, due to the ambiguities in the approval procedures for in vitro diagnostics that induce uncertainties and confusion among manufacturers. The same matter applies even more to LOCs, since new technologies may be typically more difficult to be certified.

**Figure 16:**
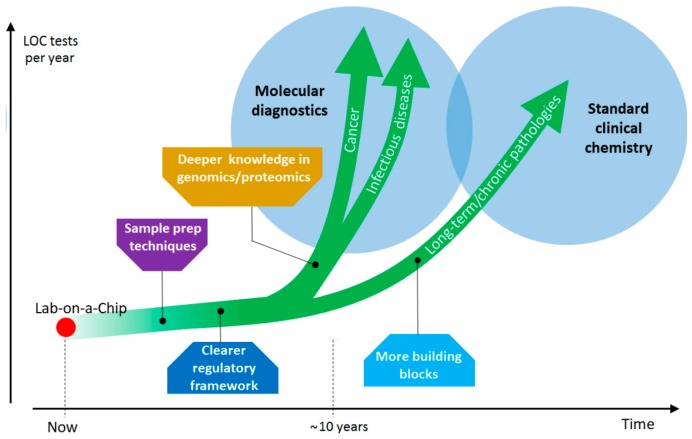
A graphical summary of the expected Lab-on-a-Chip evolution. Once sample preparation techniques have been developed and the regulatory aspects are better managed (hopefully thanks to simplification), the diffusion will rapidly grow, pervading non-hospital up to household environments, driven by important applications. In turn, specific applications will require further scientific and/or technological development, in fields where the research is very active.

Figure 16 is a graphical summary of the described perspective. The future trajectory of the red dot (LOC devices) is driven upwards (large diffusion) by a number of perspective high-impact applications (green arrows) but marked by intermediate milestones to be achieved, among wich some are more general, others are application-specific.

Overall, it is expected that a steep rise will take place, according to the perspective by which the global LOC market accounted for USD 4.23 billion in 2016 and is expected to reach USD 7.95 billion by 2022, growing at a CAGR of 11.0% meanwhile [142]. And besides these predictions, the diffusion of LOCs will deeply impact the relation between medicine and patients, considering that a large number of tests would be run on small, portable platforms, yielding fast results and requiring minimal quantities of biological sample, so being less invasive for the patient and, possibly, allowing the patients themselves to run their own analysis at home, with obvious advantages for their comfort and a reduced logistic load on hospitals.

## 8. Conclusions

In the last years, the number of POC tests is impressively increased. Most of them are based on well-established technologies such as lateral flow strips but additional advances are required in order to improve analytical performances. We expect that such improvements will continue to achieve some important issues: in particular the main desiderata for POC devices are: (1) the capability of handling small volumes of fluid; (2) milli- down to femtomolar detection sensitivity; (3) use of multiple marker panels where required; (4) low-cost and long-lasting materials, especially as disposable parts; (5) ease of use and self-containment; (6) robustness; (7) accuracy; and (8) connection through common interfaces like smartphones or personal computers. The examples in this survey witness the great and variegated effort to fulfil these needs. Examples of technologies that look promising for the future include smart contact lens sensors, able to real-time monitor the physiological parameters from tear fluid for non-invasive diagnostics [143] or tattoo-based sensors that can provide versatile tools for diagnostic purposes or body stimulation and open other interesting perspectives for POC diagnostics. Such advances can strongly affect healthy ageing and assistive technology even if there are some critical aspects that have to be overcome [144]. In conclusion, a lot of work has been done but several efforts are still necessary, but at this rate, all the indications suggest that the “lab-on-a-chip revolution” will take place within the next two decades, while pervasive improvements molecular diagnostics could even come within the next ten years.
